# Genomic analysis of TNF-related genes with prognosis and characterization of the tumor immune microenvironment in lung adenocarcinoma

**DOI:** 10.3389/fimmu.2022.993890

**Published:** 2022-11-25

**Authors:** Hua Huang, Haochuan Yu, Xuanguang Li, Yongwen Li, Guangsheng Zhu, Lianchun Su, Mingbiao Li, Chen Chen, Min Gao, Di Wu, Ruihao Zhang, Peijun Cao, Hongyu Liu, Jun Chen

**Affiliations:** ^1^ Department of Lung Cancer Surgery, Tianjin Medical University General Hospital, Tianjin, China; ^2^ Tianjin Key Laboratory of Lung Cancer Metastasis and Tumor Microenvironment, Tianjin Lung Cancer Institute, Tianjin Medical University General Hospital, Tianjin, China; ^3^ Department of Thoracic Surgery, First Affiliated Hospital, School of Medicine, Shihezi University, Shihezi, Xinjiang, China; ^4^ Department of Thoracic Surgery, the Affiliated Hospital of Inner Mongolia Medical University, Hohhot, China; ^5^ Quantitative Biomedical Research Center, Department of Population and Data Sciences, University of Texas Southwestern Medical Center, Dallas, TX, United States

**Keywords:** tumor necrosis factor, LUAD, tumor immune microenvironment, immunotherapy, prognosis

## Abstract

**Background:**

The tumor necrosis factor (TNF) family plays a role in modulating cellular functions that regulate cellular differentiation, survival, apoptosis, and especially cellular immune functions. The TNF family members also play important roles in oncogenesis and progression. However, the potential role of the TNF family members in lung adenocarcinoma (LUAD) is yet to be explored.

**Methods:**

The expression of TNF-related genes (*TNFRGs*) in 1,093 LUAD samples was investigated using The Cancer Genome Atlas and Gene Expression Omnibus datasets. The characteristic patterns of *TNFRGs* in LUAD were systematically probed and three distinct molecular subtypes were identified. Furthermore, a correlation was found between the different subtypes and their clinical characteristics. A TNF scoring system was created to predict overall survival (OS) and therapeutic responses in patients with LUAD. Subsequently, the predictive accuracy of the score was verified and a nomogram was used to optimize the clinical applicability range of the TNF score.

**Results:**

A high TNF score, involving the immune and stromal scores, indicated negative odds of OS. Moreover, the TNF score was associated with immune checkpoints and chemotherapeutic drug sensitivity. Collectively, our comprehensive *TNFRGs* analysis of patients with LUAD revealed that TNF could be involved in forming the diverse and complex tumor microenvironment, its clinicopathological features, and its prognosis.

**Conclusions:**

A TNF-related prognostic model was constructed, and a TNF score was developed. These findings are expected to improve our knowledge regarding the function of *TNFRGs* in LUAD, pave a new path for assessing the disease prognosis, and assist in developing personalized therapeutic strategies for patients with LUAD.

## Introduction

Globally, the incidence of lung cancer has increased and it is the primary cause of cancer deaths worldwide (1). Non-small cell lung cancer (NSCLC) is the primary lung cancer pathology (2), and lung adenocarcinoma (LUAD) constitutes a predominant type of NSCLC. Moreover, recently, there has been an increase in LUAD-associated morbidity (3). The survival rate of patients with LUAD remains unoptimistic, with the 5-year overall survival (OS) is about 16% (4). Immunotherapy is vital in treating advanced LUAD (5). However, immunotherapy agents are ineffective in a large number of patients (6). Therefore, it is imperative to explore effective prognostic evaluation methods and identify reliable biomarkers of patient survival to formulate highly individualized treatment and management plans for patients with LUAD.

The tumor necrosis factor (TNF) family contributes to the modulation of cellular functions involved in cellular differentiation, survival, proliferation, apoptosis, and especially immune functions against cancer cells (7). A study revealed that the members of the TNF superfamily act against pathogens and cancer cells by activating the nuclear factor-κB pathway (8). Reportedly, the success rate of immunotherapy increased by combining cytotoxic T lymphocyte antigen 4 (CTLA-4)/programmed cell death 1 (PD-1) immune checkpoint Inhibitors with the extra engagement of the TNF receptor family members (9). This implied that modulating the TNF superfamily/TNF receptor families could be a potential treatment mechanism for cancer in the future and could be applied by enhancing T-cell reactivity through engaging costimulatory receptors from the TNF superfamily/TNF receptor families. However, the specific expression modes and functions of the TNF members in LUAD remain unclarified and warrant systematic investigation. Tumor microenvironment (TME) is crucial for tumor progression (10); the density of tumor-infiltrating immune cells in the TME has been closely associated with tumor prognosis (11). Research has revealed that TNF-α induces diverse oncogenic and tumor-suppressive effects in TME (12) and that dynamic changes in TME could influence the pharmacological action of PD-1/PD ligand 1 (PD-L1) blockers, potentially developing immunotherapy tolerance (13). However, the mechanism by which TNF mediates immune cell infiltration in TME and further influences the efficacy of immunotherapy remains to be investigated.

In our study, three TNF subtypes associated with clinical consequences were established. Among the three TNF subtypes, based on the differentially expressed genes (DEGs), two gene subtypes exhibiting distinct clinical prognoses and immune cell infiltrations were identified. A scoring system to predict OS was established and used for describing the immune level of LUAD. This system may enhance the understanding of TNF in immune infiltrations and assisted in finding a new direction for more effective therapeutic strategies for LUAD.

## Materials and methods

### Data collection and processing

**Figure S1** shows a map of the process of the present work. Gene expression data, copy number variation (CNV) and somatic mutation data, and corresponding clinical information regarding LUAD were downloaded from The Cancer Genome Atlas (TCGA) database. *GSE31210* and *GSE72094* were downloaded from the Gene Expression Omnibus (GEO) database. The “Combat” algorithm was used to eliminate batch effects, and three cohorts were combined. Patients with insufficient clinicopathological or survival information were excluded from the study. Our study included 1,093 patients and their detailed clinical information is presented in **Table S1**. In total, 43 TNF-related genes (*TNFRGs*) were included in the study obtained by reviewing previous studies and sorting out the above mRNA sequencing data. The details of these *TNFRGs* are provided in **Table S2**.

### Consensus clustering analysis of tumor necrosis factor

Through “ConsensusClusterPlus” (14) package in R, unsupervised clustering analysis was applied to classify patients into different molecular subtypes based on the mRNA expression profiles of *TNFRGs.* Consensus clustering is a common research method for cancer subtype classification. Samples can be divided into several subtypes according to different sets of omics data, so as to find new disease subtypes or compare and analyze different subtypes. The distribution of the subtypes was confirmed as per the expression profiles of the genes using principal component analysis (PCA).

### Differentially expressed genes identification and functional enrichment analysis

DEGs were screened using “limma” (15) package in R among the different subtypes with a fold-change of two and an adjusted p-value <0.01. The Gene Ontology (GO) and Kyoto Gene and Genome Encyclopedia (KEGG) analyses of the DEGs were performed using “cluster profile” package in R to further explore the potential functions and enrichment pathways of the DEGs associated with different the TNF patterns.

### Construction of the tumor necrosis factor-related prognostic signature

Univariate Cox regression analysis was performed for selecting genes with prognosis value; *p* <0.05 was considered to be statistically significant. The training and test sets were randomly generated from all the patients with LUAD in a ratio of 1:1. Then, the training set was applied to establish the TNF-related prognostic signature. LASSO Cox regression analysis was used to identify the key genes and corresponding coefficients for model building. The risk score of each patient was calculated based on the standardized expression level of the key genes and their corresponding regression coefficient. The formula was established as follows: Score = GREM1 × 0.077 + GJB2 × 0.068 + CCR2 × −0.151 + MMP1 × 0.002 + IL7R × −0.006 + MS4A1 × −0.062 + HLA − DQB2 × −0.042. Patients were classified into low- and high-risk groups based on the median value of the risk score. The OS among the different groups of patients with LUAD was compared using “Survival” software package.

### Mutation and drug susceptibility analysis

The tumor mutational burden of the TCGA cohort was visualized using “maftools” (16) package in R software. “pRRophetic” (17) software package was used to calculate the half-inhibitory concentration (IC_50_) values of drugs for treating LUAD to explore the differences in drug sensitivity among the patients with different scores.

### Establishment of a nomogram scoring system

The package “rms” was utilized to construct a nomogram, providing valuable clinical predictive information regarding the clinical characteristics and risk score of patients with LUAD, particularly on 1-, 3-, and 5-year OS. In the nomogram, each clinical variable was mapped with a score and the total score was calculated by adding the scores across all the variables. Calibration plots were used to assess the predictive value among the predicted 1-, 3-, and 5-year OS and the virtually observed outcomes.

### Assessment of tumor microenvironment

Using “estimate” package, the stromal, immune, and ESTIMATE scores of each sample were computed using the ESTIMATE algorithm. The abundance of infiltrated immune cells in each sample was assessed using single-sample Gene Set Enrichment Analysis (ssGSEA).

### Statistical analysis

All statistical analyses were performed using R 4.1.2 version. The OS was compared between the different subgroups using Kaplan–Meier analysis. Time-dependent receiver operating characteristic (ROC) curve analysis was applied to assess the predictive value of the TNF score. *p* ***<*** 0.05 was considered statistically significant.

## Results

### Multiomics landscape of tumor necrosis factor-related genes in lung adenocarcinoma

**Figure 1:**
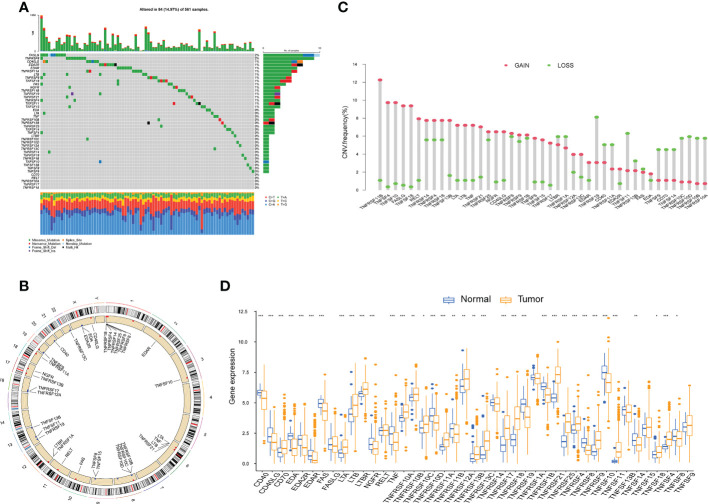
The multiomics landscape of the TNF family in LUAD. **(A)** Mutation frequency of 43 *TNFRGs* in patients with LUAD from the TCGA cohort. **(B)** Locations of CNV alterations of the *TNFRGs* on chromosomes. **(C)** Frequencies of CNV gain, loss, and nonCNV among the *TNFRGs*. **(D)** Boxplot shows the expression distributions of DEGs between LUAD and normal tissues; *p < 0.05, **p < 0.01, ***p < 0.001.

Somatic mutations in 43 *TNFRGs* involved in LUAD were observed; 84 of 561 (14.97%) LUAD samples exhibited genetic mutations. The top five mutations were *FASLG*, *TNFRSF8*, *CD40LG*, *EDA2R*, and *EDAR* (**Figure 1A**). The overall mutation frequency was low, and numerous genes had not mutated. **Figure 1B** shows the location of the CNV of these *TNFRGs* on their respective chromosomes. Among them, *TNFRSF11B*, *TNFSF4*, *TNFSF10*, *FASLG*, and *TNFRSF18* exhibited higher CNV amplification frequencies, whereas *TNFRSF19*, *TNFSF11*, and *TNFRSF10D* exhibited higher CNV deletion probabilities (**Figure 1C**). The expression levels of the 43 *TNFRGs* in tumor and normal tissues were compared; the expression levels of almost all the *TNFRGs* were significantly different between the LUAD and normal samples (**Figure 1D**). This indicates the potential role of *TNFRGs* in LUAD oncogenesis and prognosis.

### Tumor necrosis factor-related molecular patterns with distinct survival and tumor microenvironment features in lung adenocarcinoma

**Figure 2:**
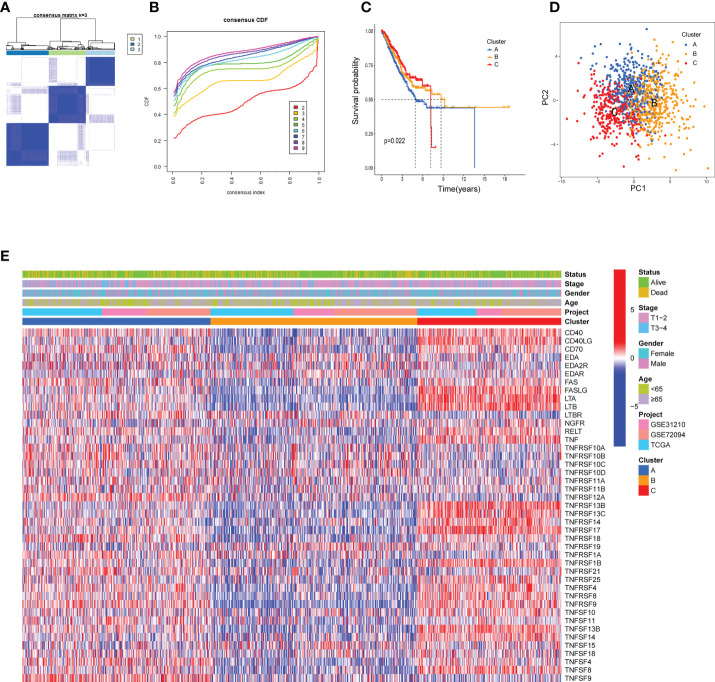
TNF clusters in the TCGA cohort. **(A)** Consensus matrix heatmap defining three clusters (k = 3) and their correlation area. **(B)** Cumulative distribution function (CDF) when k = 2–9. **(C)** Kaplan–Meier curve of the three clusters of patients with LUAD in the TCGA cohort. **(D)** PCA analysis between the three clusters. **(E)** Heatmap shows the relationships between clinicopathological characteristics of the patients and the three clusters.

The biological behaviors and expression characteristics of *TNFRGs* in LUAD were thoroughly investigated. Patients with LUAD were classified using unsupervised clustering analysis based on the expression profiles of the 43 *TNFRGs*. As a result of the consensus cumulative distribution function (CDF) curve, k = 3 was considered the optimum choice for sorting the entire cohort into subtype clusters A, B, and C (**Figures 2A, B**). The patients in cluster A displayed shorter OS time (**Figure 2C**) per the results of the Kaplan–Meier curves. PCA analysis revealed obvious distinctions in the *TNFRG* transcription profiles among the three subtypes (**Figure 2D**). The three patterns with different clinicopathological features of the patients with LUAD are illustrated in **Figure 2E**.

### Identification of tumor necrosis factor-related gene subtypes based on differentially expressed genes

**Figure 3:**
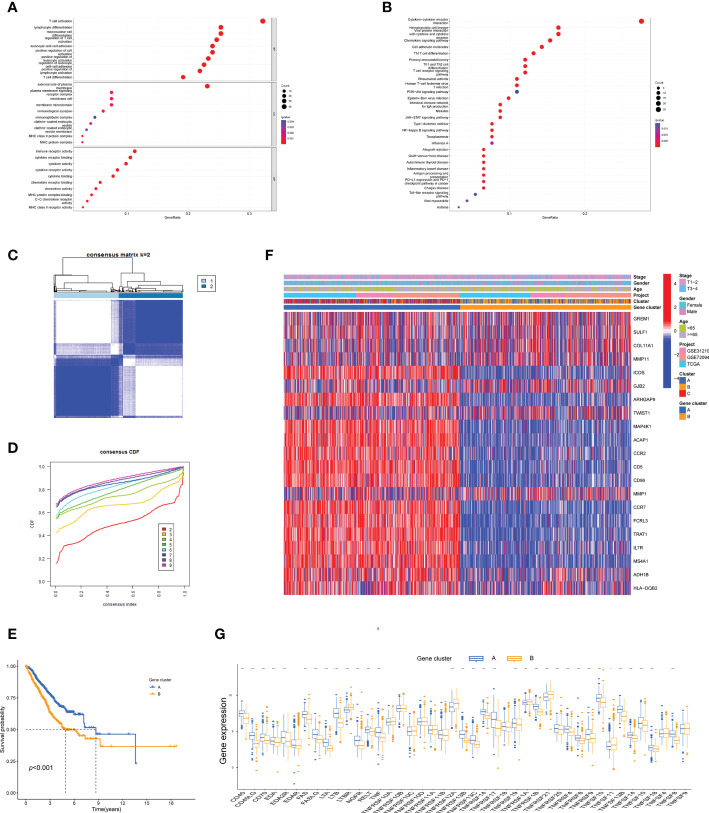
Identification of the TNF gene clusters based on the DEGs. **(A, B)** GO and KEGG enrichment among the three clusters. **(C)** Consensus matrix heatmap defining the two gene clusters (k = 2) and their correlation area. **(D)** Cumulative distribution function (CDF) when k = 2–9. **(E)** Kaplan–Meier curves for the two gene clusters (log-rank tests, *p* < 0.001). **(F)** Heatmap shows the relationships between the clinicopathological characteristics of the patients and the two distinct gene clusters. **(G)** Boxplot shows the expression distributions in 43 *TNFRGs* among the two gene clusters; *p < 0.05, ***p < 0.001.

To probe the potential biological behavior of the TNF subtypes, the TNF subtype-related DEGs were distinguished and functional enrichment analysis was conducted using R GO analysis indicated that these DEGs were significantly enriched in certain biological processes, including T-cell activation and lymphocyte differentiation (**Figure 3A**). KEGG analysis revealed immune-related enrichment pathways (**Figure 3B**). This implied that TNF acted as a critical factor in the immune regulation of the TME. Subsequently, univariate Cox regression analysis was performed to identify the genes possessing prognostic values. The patients were divided into two genomic subtypes based on prognostic genes using an unsupervised clustering analysis to further investigate the special regulation mechanism (**Figures 3C, D**). The OS time of the patients in the gene cluster A was better than those in the gene cluster B per the results of Kaplan–Meier curves (**Figure 3E**). Additionally, associations between clinicopathological features and the two gene clusters were explored, and the results indicated that most genes with prognostic values significantly differed in the A and B gene clusters (**Figure 3F**). The two *TNFRG* gene clusters demonstrated substantial distinctions in *TNFRG* expressions, as expected from the results of the TNF patterns (**Figure 3G**).

### Development and validation of tumor necrosis factor-related score

**Figure 4:**
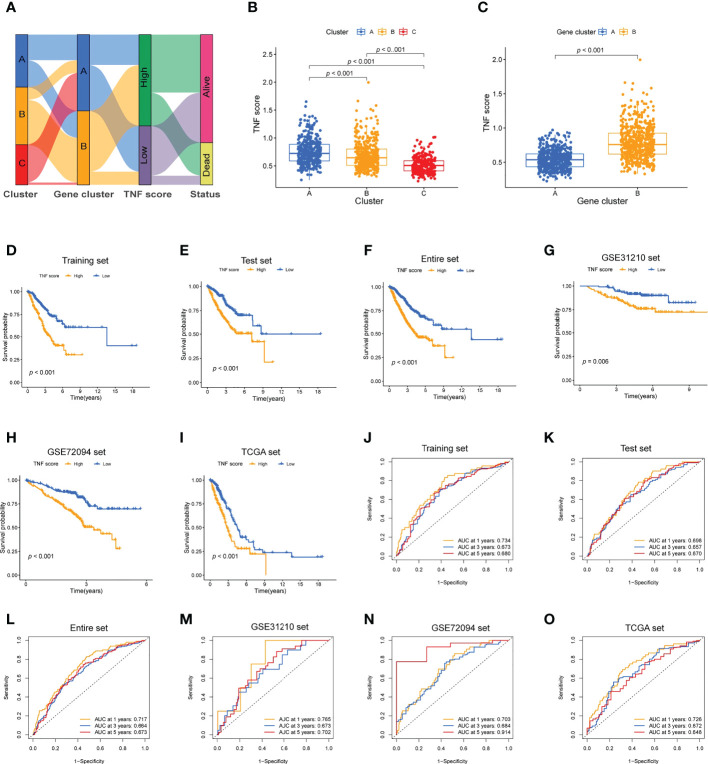
Development of the TNF scoring system and its clinical consequences. **(A)** Alluvial diagram describing the relationship of the TNF cluster, TNF gene cluster, TNF score, and survival outcome group. **(B)** Boxplot of the TNF scores among the three clusters. **(C)** Boxplot of the TNF scores between the two gene clusters. **(D–I)** Survival analysis of the patients with high and low TNF scores in the different LUAD cohorts. **(D)** Training set, *p* < 0.001; **(E)** Testing set, *p* < 0.001; **(F)** Entire set, *p* < 0.001; **(G)** GSE31210 set, *p* = 0.006; **(H)** GSE72094 set, *p* <.001; **(I)** TCGA set, *p* < 0.001. **(J–O)** Time-independent ROC analysis of the risk scores for predicting the OS.

Training and validation cohorts were randomly constructed from the included patients. LASSO Cox regression analysis was used to construct an eight-genes prognostic signature in the training cohort. The risk score of each patient with LUAD was calculated based on LASSO Cox analysis. All the patients were divided into high and low TNF score groups. The distribution of the patients and their survival outcomes in the three clusters, two gene clusters, and two risk score groups were displayed in **Figure 4A**. The patients in cluster A exhibited the highest TNF score compared with those in clusters B and C (**Figure 4B**). Meanwhile, the gene cluster B exhibited the highest TNF score (**Figure 4C**). The patients in the high TNF score group demonstrated worse OS in the training cohort (**Figure 4D**); the same observation was also noted in the test and the entire sets (**Figures 4E, F**). Next, the prognostic value of the model was validated in three independent cohorts (*TCGA*, *GSE31210*, and *GSE72094*; **Figures 4G–I**). The ROC curve further demonstrated that our model had a strong prognostic value (**Figures 4J-O**).

**Figure 5:**
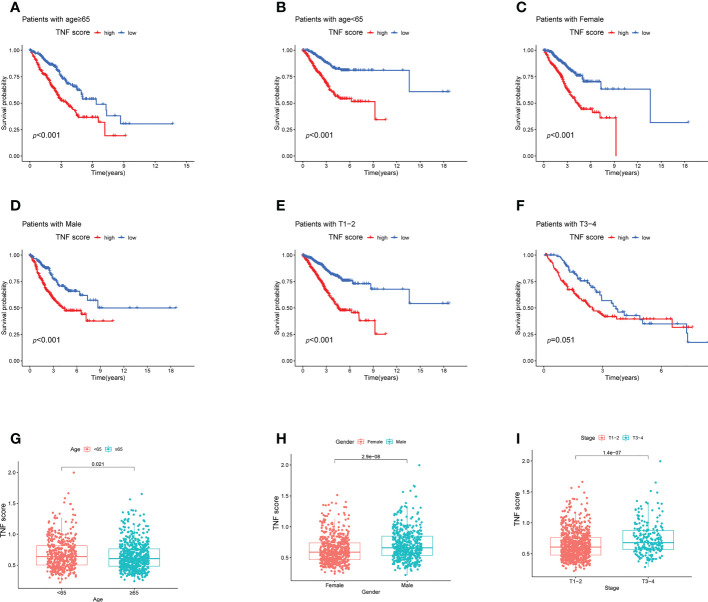
Survival analysis of the clinical stratification of OS in the LUAD cohorts. **(A–F)** Kaplan–Meier survival analysis of the high and low TNF score groups for different clinicopathological characteristics. **(A, B)** Age ≥65 or < 65 years old; **(C, D)** female or male; **(E, F)** T1–2 or T3–4. **(G–I)** Boxplot of the high and low TNF score groups for different clinicopathological characteristics. **(G)** Age <65 or ≥ 65 years old; **(H)** female or male; **(I)** T1–2 or T3–4.

Next, to verify the prognostic reliability of the different subgroups of clinical features, a detailed investigation was conducted. In the age subgroups, high-score patients were observed to exhibit a poor prognosis (**Figures 5A, B**). Similarly, in the gender (male and female) and T1–2 subgroups, in the high-score patients, a notably worse survival rate was observed (**Figures 5C–E**). Additionally, the T3–4 subgroups displayed the same trend; however, without statistical significance (**Figure 5F**; *p* = 0.051). The difference in TNF scores among age, gender, and T grade groups was also analyzed. The TNF scores in those aged <65 years, male and stage T3–4 group were significantly higher than in those aged ≥65 years, female, and stage T1–2, respectively (**Figures 5G–I**).

### Evaluation of immune infiltration and checkpoints

**Figure 6:**
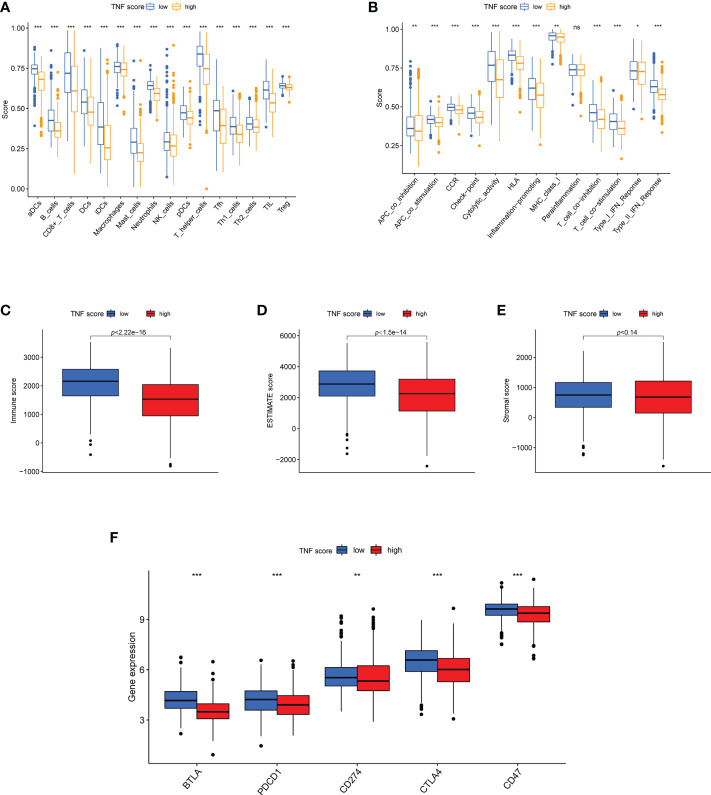
Features of TME in the high and low TNF score groups of LUAD. **(A)** The abundance of 16 infiltrating immune cell types in the high and low TNF score groups. **(B)** Correlation of the TNF scores and 13 immune functions. **(C-F)** Differences in the immune score, ESTIMATE score, stromal score and expression of five common immune checkpoints between the different TNF score groups. *p < 0.05, **p < 0.01, ***p < 0.001, and ns p > 0.05.

We further explored the association between TNF score and TME characteristics. Data revealed that the low TNF score group exhibited significantly higher scores for aDCs, B cells, iDCs, Tfh cells, T-helper cells, et al. than those exhibited by the high TNF score group (**Figure 6A**). The patients with low TNF scores exhibited significantly higher scores of HLA, cytolytic activity, and inflammation-promoting et al. (**Figure 6B**). Furthermore, the patients in the low TNF score group had distinctly higher estimate scores (**Figure 6D**; *p* = 1.5e-14) and immune scores (**Figure 6C**; *p* < 2.2e-16). However, the stromal score of the high and low TNF score groups was almost the same (**Figure 6E**; *p* = 0.14). Altogether, the TNF score exhibited a close association with TME in LUAD. Additionally, the correlations between the immune checkpoints and our risk model were investigated in detail. The results revealed that the two risk groups exhibited notably distinct immune checkpoint expressions, such as BTLA, PDCD1, CD274, CTLA4, and CD47 (**Figure 6F**).

### Construction of a prognostic nomogram and drug susceptibility analysis

**Figure 7:**
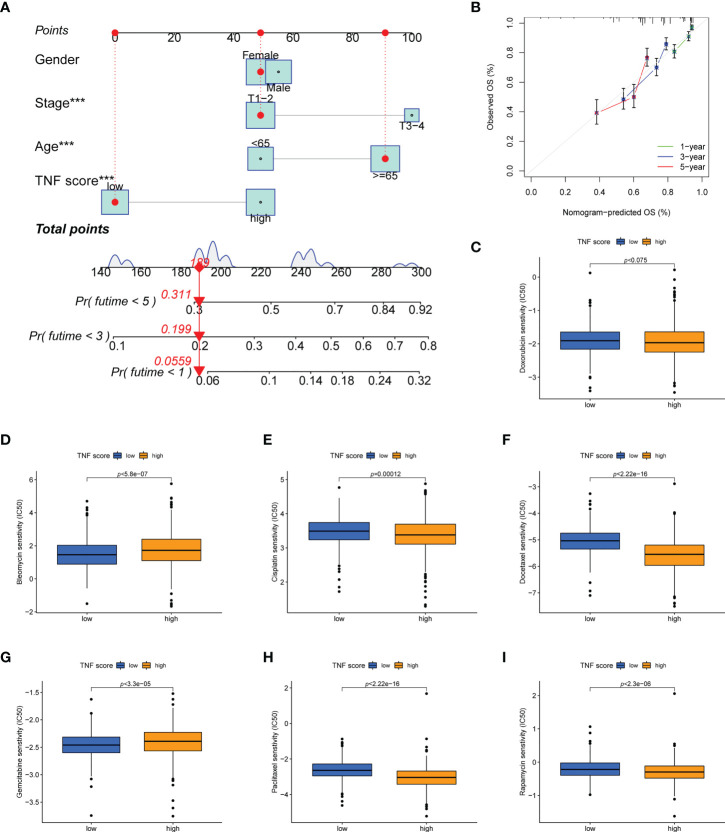
Establishment and confirmation of a nomogram. **(A)** Nomogram for predicting the 1-, 3-, and 5-year OS of patients with LUAD in the training set. **(B)** Calibration curves of the nomogram for predicting 1-, 3-, and 5-year OS in the training set. **(C–I)** Boxplots show the differences in the estimated IC_50_ levels of **(C)** doxorubicin, **(D)** bleomycin, **(E)** cisplatin, **(F)** docetaxel, **(G)** gemcitabine, **(H)** paclitaxel, and **(I)** rapamycin between TNF score and chemotherapeutic sensitivity; ***p < 0.001.

A new nomogram OS prediction model combining the TNF score and other clinicopathological parameters was developed to optimize the prediction accuracy of the risk model (**Figure 7A**). The calibration curve suggested that this nomogram was highly accurate in predicting LUAD outcomes (**Figure 7B**). We calculated the IC_50_ values of the chemotherapeutic drugs commonly applied to treat LUAD using “pRRophetic” package. The results revealed that patients with high TNF scores exhibited lower IC_50_ values for cisplatin, docetaxel, paclitaxel, and rapamycin while the IC_50_ values for bleomycin and gemcitabine were significantly lower in the patients exhibiting low TNF scores. However, the IC_50_ values of doxorubicin was not statistically different between the two groups (**Figures 7C–I**).

## Discussion

LUAD is on the verge of becoming a lung cancer type with the highest morbidity (3). Although the individualized treatment of LUAD, comprising surgery, radiotherapy, and drug therapy, has been positively developing, the prognosis of LUAD remains poor. Therefore, early prognostic indicators should direct individualized treatment and predict patient survival.

The importance of the TNF family in tumorigenesis, progression, and prognosis of various cancers is being recognized with the increased understanding of TNF. The activation of the TNF family could mediate the activation or suppression of immune response in the TME, further influencing tumorigenesis and cancer progression (18). Till date, distinct studies have suggested that the activation of CD40 is a significant mechanism in transforming so-called cold tumors into hot tumors (with prominent tumor infiltration of T cells), thereby sensitizing them to immune checkpoint Inhibitors (19). Various TNF family members, including CD40, OX40, 4-1BB, GITR, and CD27, which were investigated as new effective targets, are now under positive exploration for understanding lung cancer (20). Reportedly, dynamic changes in the TME could inhibit the pharmacological action of PD-1/PD-L1 blockers, producing immunotherapy tolerance (13). However, the manner in which TNF mediates immune cell infiltration in the TME, further influencing immunotherapy efficacy, remains to be investigated.

In our study, the clinical consequence and TME features of the TNF patterns in LUAD were amply explored. Furthermore, a *TNFRG* scoring system was established to assess the disease prognosis of individuals exhibiting different *TNFRG* mutations.

Three different molecular subtypes were identified based on the mutations in the mRNA expression profiles of *TNFRG* in patients with LUAD. The clinical prognosis of the three subtypes revealed significant differences. As per the DEGs among the three TNF subtypes, two gene subtypes associated with different clinical prognoses and immune cell infiltrations were identified. Our results revealed that *TNFRGs* might be useful for predicting the clinical prognoses and immunotherapy responses of patients with LUAD. Therefore, the effective prognostic TNF score was established for quantifying the TNF subtypes and its predictive ability was confirmed. Finally, a quantitative nomogram was established to further complement the application value of the TNF score by combining the TNF score with clinical characteristics. Our findings revealed that patients with low TNF scores exhibited a longer survival time, thereby indicating that high TNF scores could engender a worse prognosis for patients with LUAD.

Herein, *FASLG* was one of the top mutations with higher frequencies of CNV amplification compared with the rest. Previous reports have revealed that *FASLG* is important for tumorigenesis and cancer progression (21). Furthermore, our findings revealed that *TNFSF8* exhibited high mutation rate. A study reported that *TNFSF8* expression demonstrated a negative correlation with the risk of lung cancer genesis (22).

Herein, the low TNF score group exhibited higher immune infiltration than the high TNF score group. Consequently, the scores of aDCs, B cells, iDCs, Tfh cells, T-helper cells, and CD8+ cells were notably higher in the low TNF score group than in the high TNF score group. A recent study predicted the prognosis of LUAD patients, which also showed that patients with low TNF risk score showed higher immune cell infiltration, such as gamma delta T cells and macrophages M1 (23). CD8+ T cells combines with T-cell receptors and tumor cells to generate IFNg, TNF, and granzyme B and eliminate tumor cells (24). In a similar study, 12 immune cells were found to be associated with better prognosis in LUAD (25). Our results indicate that patients with low TNF scores exhibit observably higher HLA scores than patients with high TNF scores.

We found that patients exhibiting a low TNF score demonstrated higher immune and ESTIMATE scores than patients exhibiting a high TNF score. Additionally, TNF could influence tumorigenesis and cancer progression *via* TME regulation. The TME is a network system comprising cancer cells, fibroblasts, vascular cells, and inflammatory immune cells (26). Reportedly, TNF-α exhibits bidirectional effects in the TME, inducing tumorigenesis as well as tumor suppression (12).

Immunotherapy is becoming an important treatment approach for advanced LUAD. However, a large number of patients cannot benefit from PD-1/PD-L1 immune checkpoint Inhibitors owing to the defect of low universality of immunotherapy. Thus, indicating the need for another costimulatory signal of LUAD in the TME that can be examined urgently (6). In our study, as a result of analyzing the correlations between immune checkpoints and the risk model, two subtypes were found to exhibit notably different immune checkpoint expressions, such as BTLA, PDCD1, CD274, CTLA4, and CD47. A study indicated that resistance to anti-PD-1 in experimental melanoma can be eliminated by blocking the TNFa–TNFR1 axis (27). Another study indicated that delivering a high dosage of TNF into tumors is beneficial for increasing the efficacy of immunotherapy (28).

A recent study predicted the prognosis signature of Necroptosis-Related long noncoding RNA in LUAD patients (29), the AUCs of the signature in the validation cohorts were 0.609, 0.618, and 0.631 at 1, 3,5 years, respectively; in contrast, those determined by the model in the present work were 0.698, 0.657, 0.67 at 1, 3, 5 years, respectively, suggesting a slightly better predictability and stability of the proposed model.

However, this study has certain limitations. The development of multiomics profiling, distinct expression profiles, and bioinformatic methods facilitated the exploration of new prognostic models for patients with LUAD (30). However, most studies were based on entire genomic or transcriptional information from various databases and information regarding biological processes were not included. Therefore, there was an ineluctable natural bias of signatures owing to the lack of evaluation the internal characteristics of cancer in these studies. All samples included herein were based on retrospective data. Hence, large-scale experimental studies are warranted to verify the study results.

In summary, a robust and proven scoring system was established to predict OS and was used for describing the immune level of LUAD. The score was equipped to become a reliable biomarker for the survival prediction of the patients to help formulate the most individualized treatment plan. It also contributed to enhancing the understanding of TME immune infiltrations and might assist in finding a new direction for more effective immunotherapeutic strategies.

## Data availability statement

The original contributions presented in the study are included in the article/**Supplementary Material**. Further inquiries can be directed to the corresponding authors.

## Author contributions

This project was conceived by JC and HL. HH, HY, XL, and YL conducted the data analysis. GZ, LS, ML, and CC performed the data collection. MG, DW, RZ, and PC drafted the manuscript. All authors contributed to the article and approved the submitted version.

## Funding

This work was supported by the National Natural Science Foundation of China (82072595, 82172569, 81773207, and 61973232), Natural Science Foundation of Tianjin (19YFZCSY00040, and 19JCYBJC27000), Tianjin Key Medical Discipline (Specialty) Construction Project, Tianjin Health Science, and Technology Project (ZC20179).

## Conflict of interest

The authors declare that the research was conducted in the absence of any commercial or financial relationships that could be construed as a potential conflict of interest.

## Publisher’s note

All claims expressed in this article are solely those of the authors and do not necessarily represent those of their affiliated organizations, or those of the publisher, the editors and the reviewers. Any product that may be evaluated in this article, or claim that may be made by its manufacturer, is not guaranteed or endorsed by the publisher.
