# Supplementary material for: Genomic analysis of TNF-related genes with prognosis and characterization of the tumor immune microenvironment in lung adenocarcinoma
Source: Front Immunol. 2022 Nov 25;13:993890. doi: 10.3389/fimmu.2022.993890 (PMC9732939; doi:10.3389/fimmu.2022.993890)
Supplement: Supplementary file 1 [file DataSheet_1.doc]

Supplementary Material

# Supplementary Figures


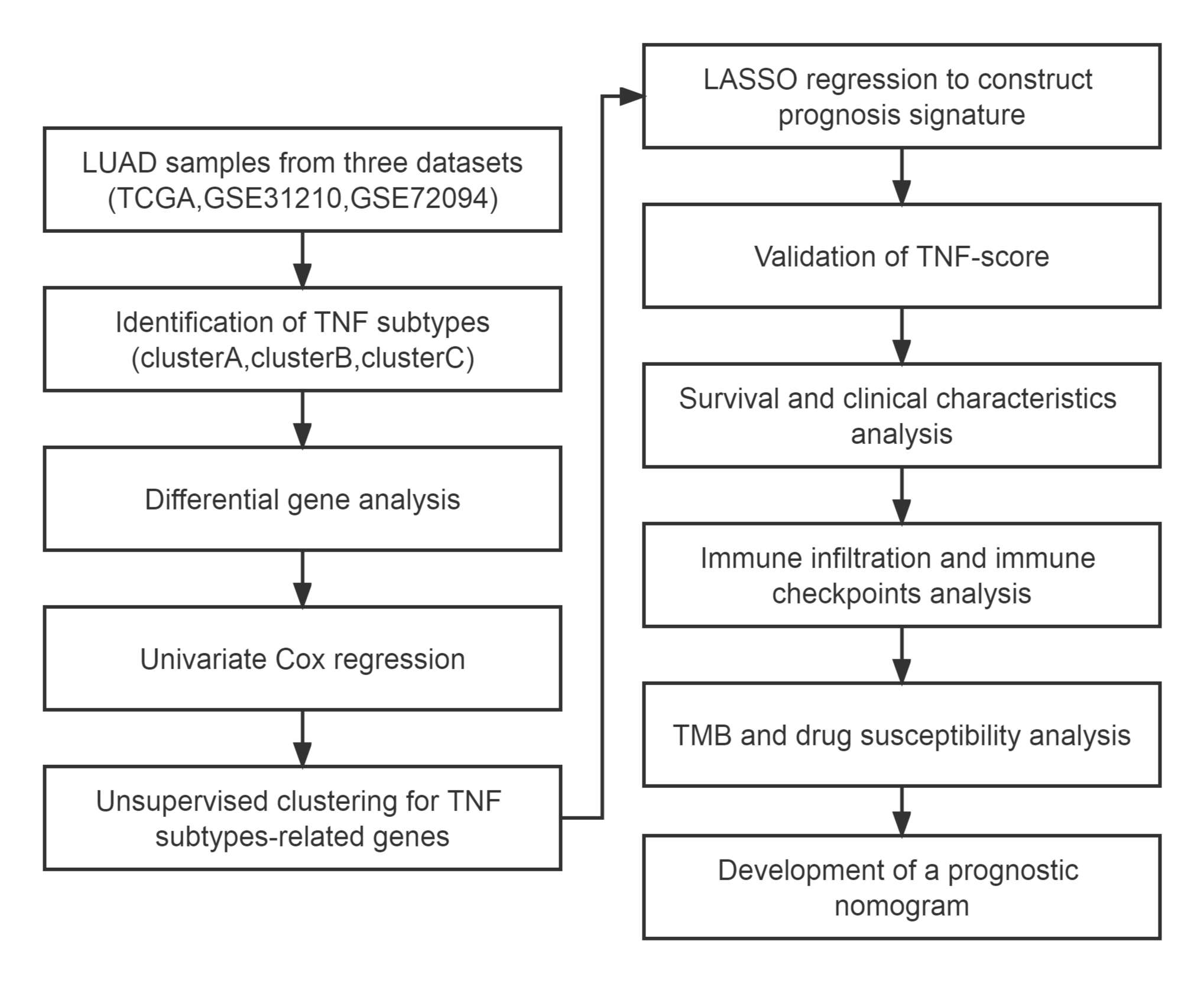


**Figure S1.** The entire analytical process of the study.

# Supplementary Tables

Table S1. The clinical characteristics of the TCGA，GSE31210 and GSE72094 cohorts.

| Clinical characteristic | | TCGA set | GSE31210 set | GSE72094 set |
| --- | --- | --- | --- | --- |
| Age | <65 | 215 | 164 | 107 |
|  | ≥65 | 254 | 62 | 291 |
| Gender | Male | 213 | 105 | 176 |
|  | Female | 256 | 121 | 222 |
| Stage | Ⅰ-Ⅱ | 367 | 226 | 321 |
|  | Ⅲ-Ⅳ | 102 | 0 | 72 |
|  | Unknown |  |  | 5 |

Table S2. Summary of TNF-related genes.

| TNF-related genes | | | | |
| --- | --- | --- | --- | --- |
| CD40 | CD40LG | CD70 | EDA | EDA2R |
| EDAR | FAS | FASLG | LTA | LTB |
| LTBR | NGFR | RELT | TNF | TNFRSF10A |
| TNFRSF10B | TNFRSF10C | TNFRSF10D | TNFRSF11A | TNFRSF11B |
| TNFRSF12A | TNFRSF13B | TNFRSF13C | TNFRSF14 | TNFRSF17 |
| TNFRSF18 | TNFRSF19 | TNFRSF1A | TNFRSF1B | TNFRSF21 |
| TNFRSF25 | TNFRSF4 | TNFRSF8 | TNFRSF9 | TNFSF10 |
| TNFSF11 | TNFSF13B | TNFSF14 | TNFSF15 | TNFSF18 |
| TNFSF4 | TNFSF8 | TNFSF9 |  |  |

Table S3. The clinical characteristics of the training and test sets.

| Clinical characteristic | | Training set | Test set | P |
| --- | --- | --- | --- | --- |
| Age | <65 | 237 | 248 | P>0.05 |
|  | ≥65 | 309 | 299 |  |
| Gender | Male | 262 | 306 | P>0.05 |
|  | Female | 284 | 241 |  |
| Stage | Ⅰ-Ⅱ | 436 | 431 | P>0.05 |
|  | Ⅲ-Ⅳ | 110 | 116 |  |
